# Two gradients, one cortex

**DOI:** 10.1093/nsr/nwag235

**Published:** 2026-04-21

**Authors:** George Paxinos, Mustafa S Kassem

**Affiliations:** Neuroscience Research Australia, Australia; School of Biomedical Sciences, The University of New South Wales, Australia; Neuroscience Research Australia, Australia; School of Biomedical Sciences, The University of New South Wales, Australia

**Figure 1.**
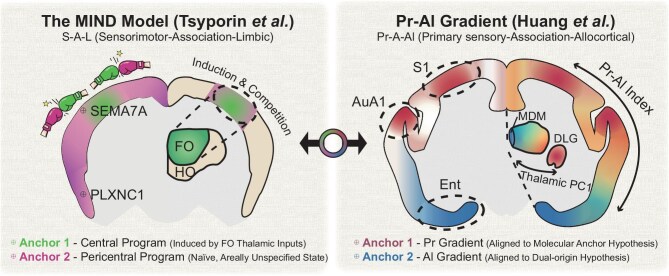
Two convergent frameworks for cortical organization. (Left) The multimodal induction−exclusion in network development model (MIND) proposed by Tsyporin *et al.* depicts the S−A−L axis as arising from competition between a central program (*SEMA7A*+, induced by first-order thalamic inputs) and a pericentral program (*PLXNC1*+, the naïve developmental default). (Right) The Pr−Al gradient proposed by Huang *et al.* shows two opposing molecular gradients radiating from primary sensory cortices (Pr pole) and allocortex (Al pole). Both models reveal parallel molecular organization in the thalamus. Thalamic PC1, the first principal component of gene expression across thalamic nuclei (Huang *et al*.), mirroring the cortical Pr−Al axis. FO, first-order nuclei; HO, higher-order nuclei; S1, primary somatosensory cortex; AuA1, primary auditory cortex; Ent, entorhinal cortex; DLG, dorsal lateral geniculate nucleus; MDM, mediodorsal nucleus.

The cerebral cortex underlies our capacity for perception, thought and voluntary action. How this structure is organized and how that organization arose through evolution has been a central question in neuroscience. Progress has been hampered by a fundamental debate: Does the cortex expand outward from ancient allocortical structures, as the dual-origin hypothesis proposes [1], or inward from early-specified sensory anchors, as the molecular anchor hypothesis proposes [2]? In a recent paper of *Science*, Huang *et al.* [3] report that these competing frameworks may describe the same organizational axis viewed from opposite ends. Combined with recent findings by Tsyporin *et al.* [4], a unified picture emerges that reframes how we think about cortical architecture (Fig. 1).

Huang *et al.* [3] integrated spatial transcriptomics with structural and functional magnetic resonance imaging (MRI) and retrograde tracing to construct the first multimodal molecular atlas of the marmoset brain. A unifying organizational principle emerged from this dataset. Two molecular gradients radiated from opposite poles: one from the primary sensory cortex (‘Pr’—visual, auditory and somatosensory areas) and the other from allocortical and periallocortical regions (‘Al’—entorhinal and piriform cortices). These gradients formed mirror images of each other (correlation coefficient *R* = −0.84), defining a continuous Pr−Al (primary sensory−association−allocortical) axis that captured the principal component of both cortical cell-type composition and gene expression. Tsyporin *et al.*, through developmental genetics, identified a sensori- motor−association−limbic (S−A−L) axis with strongly anticorrelated transcriptional programs, with *SEMA7A* marking sensorimotor territories and *PLXNC1* marking association regions.

Despite different methodologies and terminology, these studies point to the same fundamental principle. The Pr−Al axis identified by Huang *et al.* and the S−A−L axis described by Tsyporin *et al.* are functionally equivalent: primary sensory areas correspond to sensorimotor cortex and allocortical regions correspond to limbic areas (Table 1). This correspondence extends beyond terminology. Reanalysis of published data from mice, macaques and humans by Huang *et al.* revealed the same opposing Pr−Al gradients across species. Similarly, Tsyporin *et al.* demonstrated that the complementary expression pattern of *SEMA7A* and *PLXNC1* is conserved from birds to primates, with the nested organization of sensory ‘islands’ within the association cortex representing an ancient architectural motif. Together, these findings establish the opposing-gradient framework as a fundamental, evolutionarily conserved principle of cortical organization spanning at least 90 million years of mammalian evolution.

**Table 1.** Theoretical correspondence between the Pr−Al Gradient and the MIND model.

	Pr−Al gradient (Huang *et al.*) for spatial patterns	MIND model (Tsyporin *et al.*) for developmental mechanisms	Specific regions
Anchor 1	Pr gradient (primary sensory gradient)	Central program	Primary sensory areas; functionally specialized cortex with prominent layer VI (kinocortex)
Anchor 2	Al gradient (allocortical gradient)	Pericentral program	Entorhinal cortex, piriform cortex and the allocortical boundary of frontotemporal lobes
Intermediate zone	Intersection zone	Exclusion failure zone	Association cortex; regions where the two programs/gradients compete or intersect

This conserved axis exhibits dynamic developmental refinement. Huang *et al.* showed that the Pr−Al axis sharpens from birth to adulthood. Tsyporin *et al.* revealed the prenatal origin of the S−A−L axis. Gene expression shifts from fronto-temporal polarization toward the functional S−A axis during fetal development. As first-order thalamic inputs arrive, *SEMA7A* concentrates in sensory areas, while *PLXNC1* expands from fronto-temporal poles. Considered together, one study captures the prenatal induction and competition of molecular programs, while the other captures their postnatal consolidation into mature cortical architecture.

Both studies extend the gradient principle beyond cortex into subcortical structures, revealing a unified thalamocortical system. The correspondence between cortex and thalamus has long been recognized at anatomical [5], molecular [6,7] and developmental levels [8,9]. Yet how this multi-level correspondence is actively forged and evolutionarily modified remained unclear. Tsyporin *et al.* demonstrated that *SEMA7A* is highly concentrated in first-order thalamic nuclei receiving peripheral sensory input, while *PLXNC1* is highly concentrated in higher-order nuclei. Genetic deletion of cortical transcription factors *SATB2* or *ZBTB18* abolished thalamocortical projections and eliminated *SEMA7A* expression while expanding *PLXNC1* domains, demonstrating that projections to the cortex from first-order thalamic nuclei actively induce central sensory programs and exclude pericentral association programs. Huang *et al.* reported a striking molecular correspondence between cortex and thalamus: cortical Pr- and Al-highly expressed gene sets form strongly anticorrelated patterns in thalamus (*R* = −0.96), with the principal axis of thalamic gene expression (PC1) aligning with the cortical Pr−Al axis. Further, Huang *et al.* systematically expanded this analysis beyond the thalamus to other subcortical regions. They discovered that the dorsal striatum, which is a primary recipient of cortical projections, also mirrors this anticorrelation pattern. Conversely, this pronounced anticorrelation is attenuated or absent in nuclei with weaker cortical connections. Collectively, these findings demonstrate that the opposing-gradient organization is a fundamental principle specific to cortically connected systems.

The two studies offer distinct explanations for primate cortical uniqueness that operate at different temporal scales. Tsyporin *et al.* emphasize developmental heterochrony, showing that primates delay first-order thalamic neurogenesis relative to rodents, thereby granting association programs time to claim territory before sensory inputs induce focal regions, providing an elegant explanation for primate expansion. Mammals that have retained their archaic form, like hedgehogs, offer a complementary perspective. Their neocortex shows large, sharply bounded sensory areas despite diffuse thalamocortical projections [10,11]. This pattern suggests that birth-order timing may work in concert with additional processes, such as projection precision, progenitor dynamics or activity-dependent sharpening, to establish the final equilibrium. Huang *et al.* emphasize coupling strength, demonstrating that thalamocortical molecular correspondence is significantly tighter in marmosets than in mice, likely driven by the earlier arrival and prolonged interaction of thalamic axons with cortical progenitors [8,9]. This enhanced molecular coupling may facilitate coordinated gradient formation and enable more precise topographic connectivity. Together, these temporal factors—when nuclei are born and when their axons engage cortical targets—shape species-specific organization.

The convergence of these independent studies (Tsyporin *et al.* and Huang *et al.*) establishes the opposing-gradient framework as a fundamental organizing principle of the cerebral cortex. Rather than representing discrete parcels, cortical areas emerge along continuous molecular gradients anchored at sensory and allocortical poles, with association cortex occupying the intersection zone. This framework reshapes our understanding at multiple levels. For cortical parcellation, it suggests that sharp molecular boundaries arise where opposing gradients intersect, providing a mechanistic basis for area specification beyond traditional cytoarchitectonic criteria. For brain networks, the gradient intersection zones where the default mode network and frontal pole reside represent convergence hubs for multimodal sensory streams, potentially explaining both their role in abstract cognition and their vulnerability to neuropsychiatric disorders. For evolution, the framework reveals how a conserved organizational principle can generate diverse cortical landscapes. Modulating thalamic nuclei timing or thalamocortical coupling can shift the sensorimotor−association equilibrium. Ultimately, the opposing-gradient pattern offers a unifying framework that connects molecular mechanisms, developmental processes, functional architecture, evolutionary trajectories and disease vulnerabilities. The cortex, far from being a patchwork of disconnected regions, is fundamentally organized along an axis that defines both its structure and its capacity for adaptive specialization.
